# ALOX15‐Mediated Neuron Ferroptosis Was Involved in Diabetic Peripheral Neuropathic Pain

**DOI:** 10.1111/cns.70440

**Published:** 2025-05-19

**Authors:** Zhiye Feng, Fuye Li, Zhiqiang Lin, Jian Liu, Xi Chen, Wenxu Yan, Zhongjie Liu

**Affiliations:** ^1^ Department of Anesthesiology, Zhujiang Hospital Southern Medical University Guangzhou China; ^2^ Department of Critical Care Medicine, Nanfang Hospital Southern Medical University Guangzhou China; ^3^ Zhongshan Hospital of Traditional Chinese Medicine Zhongshan China; ^4^ Shenzhen LuoHu People's Hospital Shenzhen China; ^5^ Department of Anesthesiology Shenzhen Children's Hospital Shenzhen China

**Keywords:** ALOX15, diabetic peripheral neuropathic pain, ferroptosis, machine learning

## Abstract

**Background:**

Diabetic peripheral neuropathic pain (DPNP) is one of the most common complications in diabetic patients. Current treatment strategies primarily focus on blood glucose control and pain relief, but they often yield limited effects. Ferroptosis, a regulated form of cell death driven by lipid peroxidation and iron imbalance, plays a crucial role in various diseases, including neuropathic pain.

**Methods:**

In this study, we employed a combined bioinformatics and machine learning approach to identify genes most strongly associated with DPNP and ferroptosis. Subsequently, we established a DPNP mouse model via streptozotocin (STZ) injection and a high‐glucose‐induced SH‐SY5Y cell injury model. ALOX15 was knocked down in the in vitro model using siRNA transfection.

**Results:**

Bioinformatics analysis identified ALOX15 as a hub gene linking DPNP and ferroptosis. In both in vivo and in vitro DPNP models, ALOX15 expression was significantly upregulated and correlated with ferroptosis biomarkers. Knockdown of ALOX15 in the cellular model mitigated high‐glucose‐induced ferroptosis, reduced lipid peroxidation and free iron ion accumulation, and restored cell viability.

**Conclusion:**

In conclusion, ALOX15 contributes to the onset and progression of DPNP by promoting ferroptosis, and its knockdown effectively suppresses ferroptosis, providing a novel target and strategy for DPNP treatment.

## Graphical abstract

High glucose levels may enhance the peroxidation of PUAFs in neurons by upregulating the expression of ALOX15. Concurrently, it inhibits GPX4 antioxidant enzymes and FTH1 molecules, leading to an increase in intracellular Fe^2+^, which promotes the production of lipid ROS through the Fenton reaction, mediating ferroptosis in neurons and ultimately contributing to DPNP.

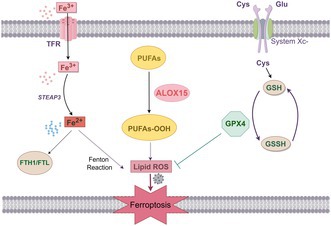

## 1 Introduction

In 2021, the International Diabetes Federation stated that 536.6 million adults worldwide had diabetes. Diabetic peripheral neuropathic pain (DPNP) is a common complication in diabetic patients, with as many as 50% of diabetic patients developing neuropathy over the course of their disease, and 50% experiencing neuropathic pain [1, 2]. This pain is considered one of the most challenging to treat, and current treatments, which focus on glucose lowering and pain relief, are often ineffective [3]. At the same time, the underlying molecular mechanisms of DPNP development remain unknown, hindering the development of targeted therapies.

Ferroptosis, a recently recognized mode of cell death, is characterized by increased ferrous ions and the accumulation of lethal lipid ROS (Reactive Oxygen Species) [4, 5]. To date, not only has ferroptosis been found to play an important role in tumors, diabetes and its many complications, and neuropathic pain, but studies have also shown that attenuating ferroptosis attenuates disease progression [6, 7, 8, 9, 10, 11, 12, 13]. Therefore, pharmacological modulation of ferroptosis is a promising therapeutic strategy for DPNP [14].

Arachidonate 15‐lipoxygenase (ALOX15), a key enzyme mediating ferroptosis, catalyzes the peroxidation of polyunsaturated fatty acids (PUFAs) such as arachidonic acid (AA) and linoleic acid (LA), thereby driving ferroptosis [15]. By combining bioinformatics and machine learning methods, we identified ALOX15 as a key candidate gene for regulating DPNP in ferroptosis. Although previous studies suggested that ALOX15 might be involved in the regulation of neuropathic pain [10, 16], the involvement of ferroptosis in the pathogenesis of DPNP has not been reported in the literature; moreover, the functional role of ALOX15 in the ferroptosis pathway of DPNP remains unclear.

Building on these findings, we employed a DPNP murine model and a high glucose (HG)‐induced cellular injury model to investigate the pathogenesis of DPNP. We observed significant upregulation of ALOX15 in both in vivo and in vitro DPNP models, with its expression correlating with ferroptosis biomarkers. Furthermore, through the ALOX15 gene silencing, we discovered that the knockdown of ALOX15 could alleviate ferroptosis induced by HG, reduce lipid peroxidation, and restore cell viability. This study aims to reveal the connection between ferroptosis and DPNP, and clarify the role of ALOX15 in ferroptosis within the DPNP model, thereby identifying a novel therapeutic target for precision treatment of DPNP.

## 2 Materials and Methods

### 2.1 Animals

All animal studies were conducted in accordance with the *Guide for the Care and Use of Laboratory Animals* (NRC 1996) and were approved by the Animal Ethics Committee of Zhujiang Hospital, Southern Medical University (Protocol Number: LAEC‐2023‐035, Guangzhou, China). Male C57Bl/6j mice were purchased from Zhuhai BesTest Bio‐Tech Co. Ltd. All animals were housed under specific pathogen‐free (SPF) conditions (23°C–24°C, 40%–60% relative humidity, 12/12‐h light/dark cycle) with free access to food and water.

### 2.2 Establishment of the DPNP Model

Streptozotocin (STZ) is widely used to create animal models of type 1 and type 2 diabetes. Based on previous research methods [17], we established a DPNP mouse model by intraperitoneally injecting 50 mg/kg of STZ (Sigma‐Aldrich) into 5‐week‐old mice for 5 consecutive days to induce diabetes. Mice were then screened for the T1DM‐related DPNP model by weekly monitoring of blood glucose levels, paw withdrawal threshold (PWT), and paw withdrawal latency (PWL). Mice exhibiting a significant reduction in PWT and PWL after 10 weeks of age were considered DPNP model mice. Subsequently, L4‐L6 DRG tissues were harvested for Western blot analysis.

### 2.3 Von Frey Filament Test for Mechanical Allodynia

Mice were placed on a testing metal mesh and allowed to acclimate until they were calm. Using the Up‐and‐Down method, Von Frey filaments with varying forces were applied to the plantar surface of the hind paw to deliver point stimuli, and the PWT was recorded.

### 2.4 Measurement of Thermal PWL Using Hargreaves Apparatus

An infrared light source was positioned beneath a glass plate. During testing, the light source was slowly moved to align with the center of the mouse's hind paw. When the photodetector sensed the hind paw lifting off the glass plate, the heat source automatically turned off and the timer stopped. A positive response was recorded when the mouse lifted or licked its paw, and the time was noted. Each measurement was separated by at least 5 min, and each hind paw was tested three times per mouse. The average of these three measurements was calculated as the mouse's thermal PWL. A cutoff time of 20 s was set; if no response occurred within this period, heating was automatically terminated to prevent thermal injury to the paw.

### 2.5 Data Collection and Analysis and Gene Set Enrichment Analysis

Retrieved from the GEO database, the expression profiling dataset GSE34000 contains data from the dorsal root ganglia (DRG) of STZ‐induced diabetic painful neuropathy rats, comprising 3 DPNP rats and 3 normal control rats.

Differential expression analysis was performed using the R software “DESeq2 (v1.4.5)” package, where differentially expressed genes were selected using the filtering criteria of |log2FC| > 0.58 and *p* < 0.05. The “ggplot2” and “heatmap” packages in R software were used to visualize the data.

The database of ferroptosis genes is FerrDb (FerrDb(zhounan.org)). In addition, we used the CIBERSORT algorithm [18] to analyze the immune infiltration pattern.

To investigate potential mechanisms between the treatment and control groups, the “mh.all.v2022.1.Mm.symbols.gmt” file was downloaded from the Molecular Signature Database (GSEA|MSigDB(gsea‐msigdb.org)) [19] and analyzed using the “clusterProfiler” R package [20] for Gene Set Enrichment Analysis (GSEA). Displayed were the top three pathways with the greatest enrichment, with a *p* < 0.05 indicating statistical significance.

### 2.6 Protein–Protein Interaction

The protein–protein interaction (PPI) network among intersecting genes was constructed using the GeneMANIA website (GeneMANIA) [21]. GeneMANIA aids in predicting the functions of genes and genomes by utilizing functional association datasets to identify genes related to the input genes.

### 2.7 Identification of Hub Genes

In addition, to select potential hub genes, the random forest (RF) algorithm was applied. A RF model was constructed with differentially expressed genes being the dependent variable while diseases the independent variables. After parameter tuning, the model was optimal when the number of decision trees (ntree) included in the RF model was set to 500 and the count of variables (mtry) included in each decision tree was set to 7. The results showed that ALOX15 was the highest ranked of the intersecting genes, and thus was considered a ferroptosis‐related hub gene in DPNP.

### 2.8 Cell Culture and Treatment

SH‐SY5Y cells (SH‐SY5Y, SCSP‐5014), an undifferentiated human neuroblastoma cell line purchased from the cell bank of the Shanghai Institutes for Life Sciences, Chinese Academy of Sciences. In previous studies, SH‐SY5Y cells have often been used as the research model for neuronal injury [22, 23, 24]. SH‐SY5Y cells were cultured in MEM/F12 medium containing 10% fetal bovine serum and 1% penicillin–streptomycin (Gibco, Invitrogen Life Technology, Carlsbad, California, USA) at 37°C in a 5% CO_2_ atmosphere. The medium was changed every 2 days, with cell passaging occurring every 4 days. When the cells were treated with different concentrations of glucose, we adjusted the glucose concentration in MEM/F12 medium by adding glucose via a sterile filter, accounting for the baseline glucose content of the medium.

### 2.9 Cell Viability Assay

Cell viability was measured using the Cell Counting Kit‐8 (CCK‐8) assay. SH‐SY5Y cells were inoculated in 96‐well plates at a concentration of 1 × 10^4^ cells/well. Ten microliters of CCK‐8 reagent (Yiyuan Biotechnology, Guangzhou, China) was added to 96‐well plates and incubated for 2 h. The cell viability was measured using an enzyme marker. Absorbance was read at 450 nm with a microplate reader (Bio‐Rad, CA, USA).

### 2.10 Gene Knockdown by siRNA

SH‐SY5Y cells were inoculated in 6‐well plates at a density of 1 × 10^6^/well and transfected with siRNA oligonucleotides targeting ALOX15 (Gemma Genetics, Shanghai, China) using Lipofectamine 3000 Transfection Reagent (L3000015, Invitrogen) for 8 h. Cells were incubated with MEM/F12 medium for 24 h. Serving as the negative control was si‐NC. The effectiveness of siRNA was confirmed by using qRT‐PCR to assess the knockdown efficiency of each sequence, and then the sequence with the highest efficiency was selected for transfection. Western blotting (WB) detected ALOX15 protein expression levels.

The si‐RNA nucleotide sequences are as follows:

si‐NC, sense (5′–3′): UUCUCCGAACGUGUCACGUTT,

antisense (5′–3′): ACGUGACACGUUCGGAGAATT;

si‐ALOX15(1), sense (5′–3′): S: GACGGGGUUAAUUCUGAAUATT,

antisense (5′–3′): UAUUCAGAAUUAACCCGUCTT;

si‐ALOX15(2), sense (5′–3′): S: GCGAUACACCCUGGAAAUUTT,

antisense (5′–3′): AAUUUUCCAGGGGUGUGUUAUCGCTT;

si‐ALOX15(3), sense (5′–3′): GGUGGAAGUACCGGAGUAUTT,

antisense (5′–3′): AUACUCCGGUACUUCCACCTT.

### 2.11 RNA Extraction and Analysis by qRT‐PCR

RNA was extracted with the TRIzol kit (Invitrogen) and reverse transcribed to cDNA using the qRT‐PCR kit (Invitrogen) following the manufacturer's instructions. The synthesized cDNA was amplified on a LightCycler 480 (Roche, Germany). Each qRT‐PCR assay used three biological replicates. The expression level of β‐actin was determined using the $2^{-\Delta \Delta C_{T}}$ method. The following forward and reverse primer sets were used:

R‐β‐actin‐F: GAAGGTCGGGTGTGAACGGAT.

R‐β‐actin‐R: CCCATTTGATGTTAGCGGGGAT.

R‐Alox15‐F: CTTAAGGACGACGCCTGGTT.

R‐Alox15‐R: GCGGTAACAAGGGAACCTGA.

### 2.12 Western Blotting

WB was performed to detect ALOX15, GPX4, FTH1, and 4HNE protein levels. Cells were collected, and protein lysates prepared. Afterward, protein samples were isolated using SDS‐PAGE and transferred to a 0.22 μm PVDF membrane. The membrane was blocked using fast blocking western solution for 10 min and then treated with primary antibodies, including anti‐ALOX15 (1:500, ab244205, Abcam), anti‐GPX4 (1:500, T56959F, abmart), anti‐FTH1 (1:500, YT1692, ImmunoWay), anti‐4HNE (1:500, SMC‐511D, StressMarq), and anti‐β‐actin (1:40,000, RM2001, Ray Antibody Biotech) overnight at 4°C. After washing, membranes were incubated with HRP‐conjugated secondary antibodies, and bands were visualized using ECL chemiluminescence. The density of protein bands was examined using NIHA ImageJ software.

### 2.13 Immunodeficiency

Cells were cultured in confocal petri dishes and then treated according to grouping requirements. Cells were fixed with 4% paraformaldehyde (MACKLIN, Shanghai, China) for 20 min at room temperature and permeabilized with 0.3% PBST (PBS + Triton X‐100) for 20 min. Thereafter, cells were blocked with 5% BSA for 1 h and incubated with primary antibody MDA (1:500, ab243066, Abcam) overnight at 4°C. After washing with PBS, cells were incubated with fluorescent secondary antibody (1:1000, ab150105, Abcam ab8135) for 1 h at 37°C. Cell nuclei were stained with 4′,6‐diamidino‐2‐phenylindole (DAPI; CST, Shanghai, China) for 10 min. A Nikon laser confocal microscope was used to take photographs to capture the final images. Image J software is used for quantitative analysis by removing the background and selecting the cells, and calculating the mean fluorescence intensity of at least 20 cells from 3 random images.

### 2.14 Flow Cytometry

After treatment, cells were washed with PBS (2 mL) and incubated with 0.25% trypsin (0.5 mL) for 1 min at 37°C in an incubator with 5% CO_2_. The cell suspension was transferred from the plate to a centrifuge tube. After centrifugation for 3 min and removal of the supernatant, cells were resuspended by adding 0.5 mL of PBS for flow cytometry (BD Biosciences, Franklin Lakes, NJ, USA). FlowJo and GraphPad Prism (version 9) software were used to analyze results.

### 2.15 Lipid Peroxidation

C11‐BODIPY581/591 was used to quantify the level of oxidized lipids in SH‐SY5Y cells. First, cells were incubated with the working solution (10 μM) for 30 min. After incubation, the cells were washed three times to remove the medium. Fluorescence was detected by flow cytometry (FITC channel). C‐11 BODIPY fluorescence was observed with a Nikon laser confocal microscopy. Lipid oxidation levels were quantified through measuring fluorescence intensity and calculating the ratio of fluorescence measured under the FITC (510 nm) and Texas Red (590 nm) channels [25].

### 2.16 Fe^2+^ Content

Cells were washed three times with PBS and then incubated with a working solution (1 μM) prepared using MEM/F12 without FBS for 30 min in an incubator. Fluorescence was detected by flow cytometry (FITC channel). FerroOrange fluorescence was observed with a Nikon laser confocal microscope.

## 3 Statistical Analysis

Bioinformatics analysis was performed using RStudio (version 4.2.3). Statistical analysis was performed using GraphPad Prism version 9 (GraphPad Software, San Diego, CA, USA) and ImageJ software (version 1.8.0.172). The normality of data was checked with the Shapiro–Wilk normality test (*α* = 0.05) before analysis. A two‐tailed unpaired Student's *t*‐test was used to assess the differences between the two groups. To analyze multiple group differences, Tukey's multiple comparisons using multiple sample means compared two‐by‐two was followed. The data is represented as the mean ± standard deviation (SD).

## 4 Result

### 4.1 Differential Gene Expression Analysis and GSEA, Alox15 Upregulated in DRG of DPNP Rats

**FIGURE 1:**
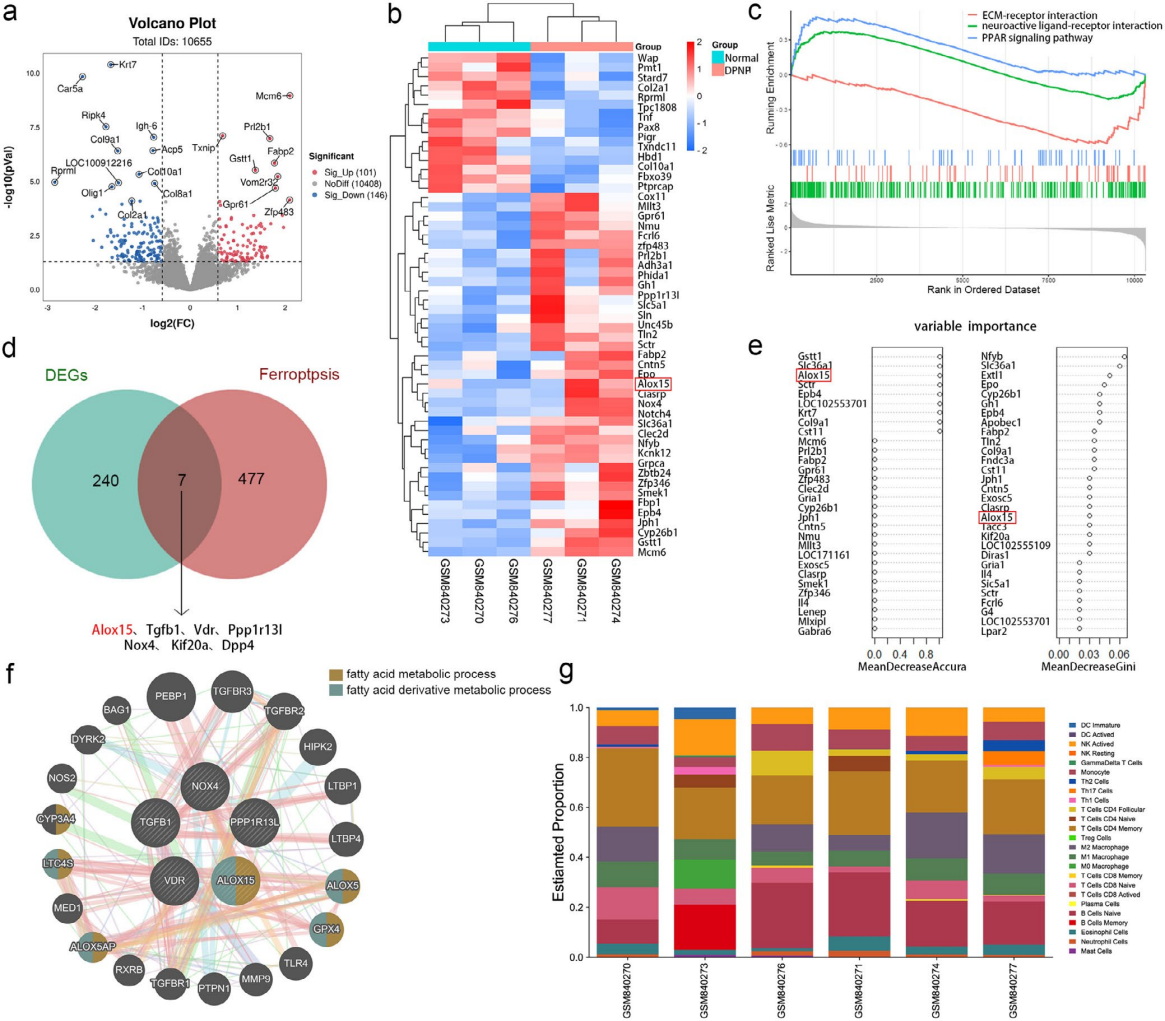
Differential gene expression analysis and GSEA, ALOX15 upregulated in DRG of DPNP rats. (a) Volcano plot showing upregulated and downregulated genes. (b) Heat map of the 50 most differentially expressed genes regulated by DPNP. Expression down‐regulation is shown in blue, whereas up‐regulation is represented in red. (c) The three most differentially expressed pathways in GSEA. (d) Venn diagram of differentially expressed genes and ferroptosis‐related genes. The Venn diagram shows that the intersection includes seven genes, demonstrating that they are all under the regulation of DPNP as well as implicated in ferroptosis. (e) Importance ranking of the random forest algorithm differentially expressed genes. (f) The GeneMANIA website is used to establish the PPI network. The 20 functionally similar genes are located in the outer circle, while the intersection genes are located in the inner circle. (g) The proportion of immune cells was shown.

Based on∣log2FC∣ > 0.58 and a *p* < 0.05, 247 differentially expressed genes were found, comprising 101 genes that were up‐regulated and 146 genes that were down‐regulated. The complete table can be found in Supporting Information S1. Volcano plots presented the differences between up‐regulated and down‐regulated genes (Figure 1a). Next, the top 50 differentially expressed genes were visualized through heatmap analysis, and we found that the Alox15 gene of the ferroptosis pathway was upregulated in the DPNP group (Figure 1b). To elucidate the potential regulatory mechanisms of DPNP, GSEA analysis was performed. Gene sets showed significant differences in pathways such as ECM‐receptor interaction, neuroactive ligand‐receptor interaction, and PPAR signaling pathway (Figure 1c).

By intersecting differentially expressed genes with ferroptosis‐related genes, a Venn diagram revealed 7 intersecting genes, suggesting that these genes are regulated by DPNP and involved in ferroptosis (Figure 1d).

To identify key genes, we used GeneMANIA to perform PPI analysis on the intersecting genes. The intersecting genes were positioned in the inner circle, while the predicted genes were in the outer circle (Figure 1f). The results showed that only ALOX15 and GPX4 were connected. Their functions are primarily concentrated in the fatty acid metabolic process and the fatty acid derivative metabolic process, which align with the ferroptosis pathway, where lipid peroxides are the ultimate executors of ferroptosis. It has been reported that, in the context of diabetes, inflammation mediated by memory T cells can exacerbate nerve pain and nerve damage in patients with DPNP [26]. As shown in the proportion of immune cells, CD4^+^ Memory T Cells accounted for the largest proportion (Figure 1g). Immune infiltration analysis was conducted to evaluate the presence and distribution of immune cells in tissues and to understand their potential influence on the development and treatment of DPNP.

Our previous research suggested a close relationship between ferroptosis and DPNP, and ALOX15 was one of the upregulated genes associated with the ferroptosis pathway. Given that ALOX15 ranked highest in importance among 7 intersecting genes according to the RF algorithm (Figure 1e), and that ALOX15 was the only gene connected to GPX4 (key regulator of ferroptosis) in the PPI network, ALOX15 was identified as a key gene in DPNP–ferroptosis.

### 4.2 Construction of DPNP Model; Increased Expression of ALOX15 and Ferroptosis‐Related Proteins in DRG of DPNP Mice

**FIGURE 2:**
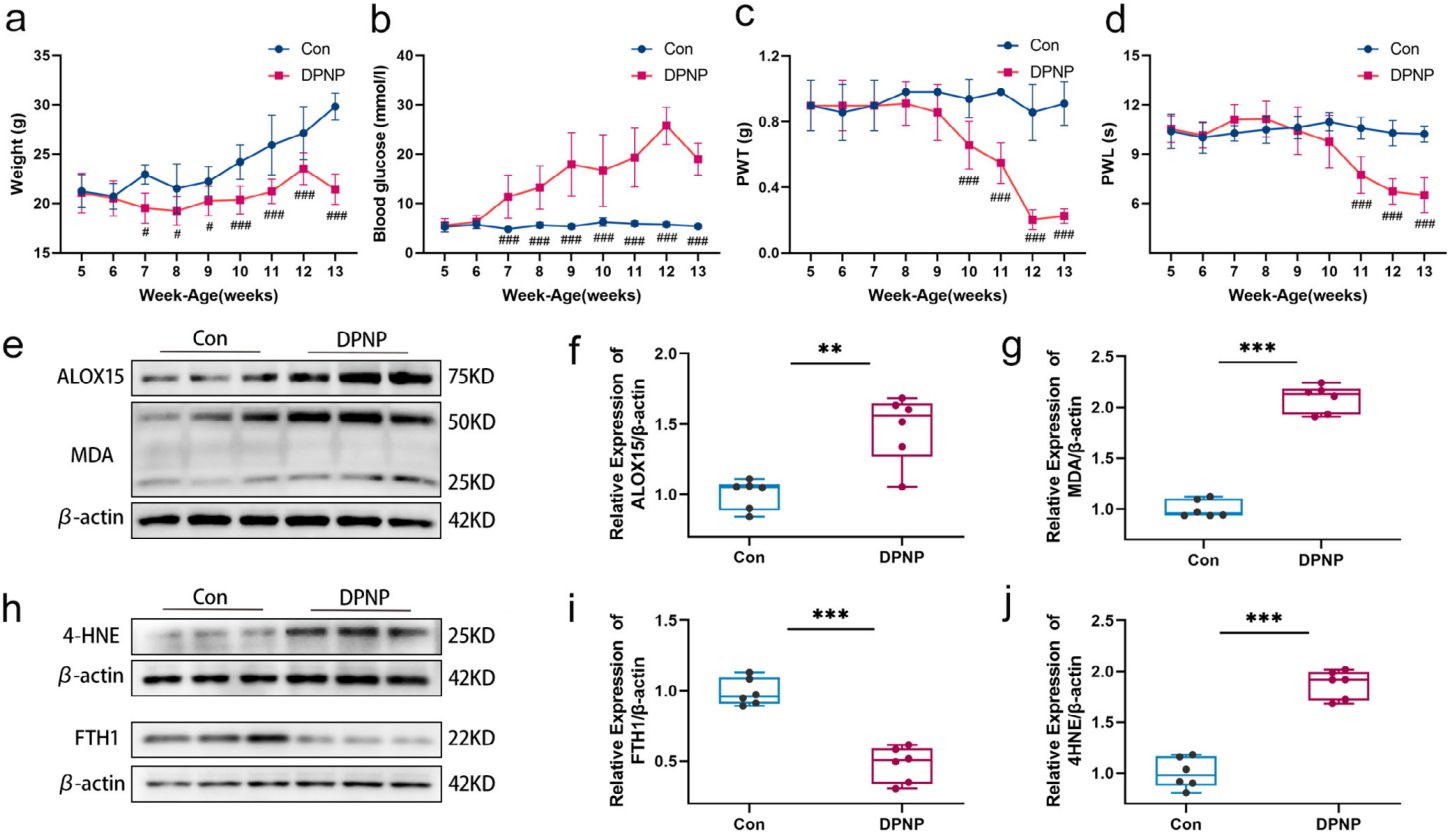
Construction of DPNP model; increased expression of ALOX15 and ferroptosis‐related proteins in DRG of DPNP mice. (a–d) Time course of body weight (a), blood glucose (b), PWT (c) and PWL (d) in the DPNP and control mice. After 10 weeks, the mice in the DPNP group showed obvious mechanical pain and thermal pain hypersensitivity. (e–j) Protein expression levels of ALOX15, MDA, 4HNE, and FTH1. Two‐tailed unpaired student *T*‐test, *n* = 6 mice/group. Data are presented as mean ± standard deviation. ^#^ *p* < 0.05, ^###^ *p* < 0.001, **p < 0.01, ***p < 0.001. PWL, paw withdrawal latency; PWT, paw withdrawal threshold.

A DPNP model was established in mice to determine pathological changes in the DRG. Body weight, blood glucose, PWT, and PWL were measured weekly. Compared to control mice, DPNP mice exhibited a significant decrease in body weight and a significant increase in blood glucose levels (Figure 2a,b), indicating that a typical type 1 diabetes mellitus (T1DM) mouse model was used in this study. Mice with significantly reduced PWT and PWL after 10 weeks of age were DPNP models (Figure 2c,d). Western Blot analysis was performed on L4‐L6 DRG tissues.

Malondialdehyde (MDA) and 4‐hydroxynonenal (4‐HNE) are markers of lipid peroxidation and cytotoxicity; their levels reflect the degree of lipid peroxidation. It is well known that ferritin heavy chain 1 (FTH1) functions in iron storage, and downregulation of FTH1 expression can increase intracellular Fe^2+^ concentration, promoting ferroptosis. The results showed that with the overactivation of ALOX15, MDA and 4‐HNE expressions in the DRG were upregulated, while FTH1 expression was suppressed (Figure 2e–j). This confirms that lipid oxidation levels are elevated and iron homeostasis is disrupted in the DRG of DPNP mice, suggesting that the overactivation of ALOX15 may be associated with ferroptosis.

### 4.3 ALOX15 Expression Was Increased in HG Injury Cell Model

**FIGURE 3:**
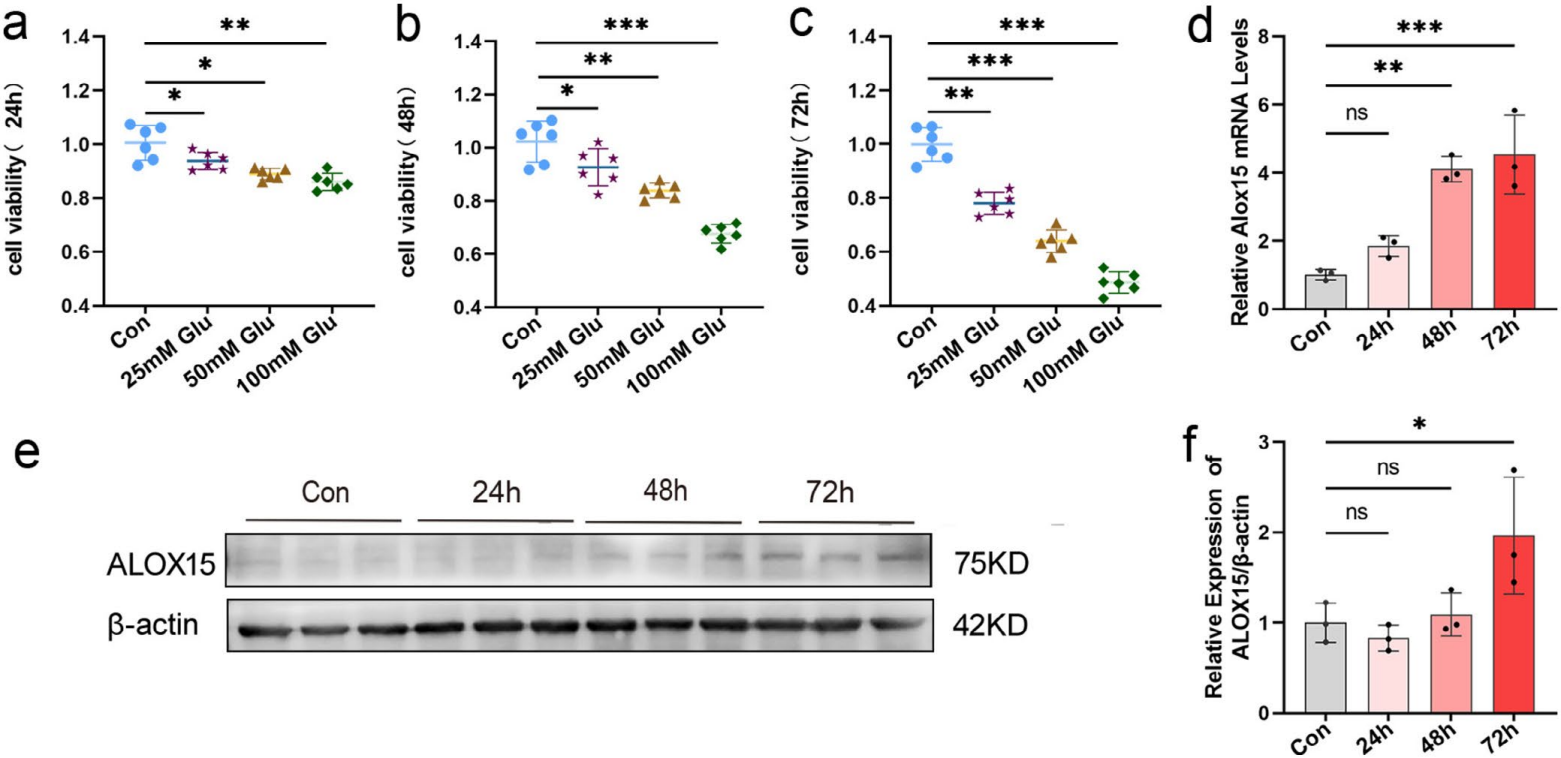
ALOX15 expression was increased in high glucose injury cell model. (a–c) Trends in cell viability of neuronal cells treated with different glucose concentrations for 24, 48, and 72 h, respectively. (d) ALOX15 expression was analyzed through qPCR, with mRNA levels normalized to β‐actin. (e) WB of ALOX15 with 50 mM glucose. (f) Quantitative analysis of ALOX15 expression, with levels of expression normalized to β‐actin. Data are shown as mean ± SD. One‐way ANOVA was used, followed by Tukey's multiple comparisons using multiple sample means compared two‐by‐two. *p < 0.05, **p < 0.01, ***p < 0.001. *n* = 3–6 independent cell culture preparations.

To simulate and study the pathogenesis of DPNP, we selected a HG injury cell model. The effect of HG on neuronal cell viability was first examined using a CCK‐8 assay, which indicated that glucose in SH‐SY5Y cells escalated over both the duration of exposure and concentration (Figure 3a–c). Based on the CCK‐8 results, under the treatment of 50 mmol/L glucose concentration, the inhibition rate of cell viability was close to 60%. We chose this concentration as the subsequent intervention treatment.

Next, we used qPCR and WB to validate the RNA‐seq outcomes. First, we examined the transcriptional and translational levels of ALOX15 in SH‐SY5Y cells treated with 50 mM glucose for different durations. Both qPCR and WB analyses indicated that ALOX15 expression was significantly elevated in the HG group compared to the control group (Figure 3d–f). These findings confirmed that HG inhibits SH‐SY5Y cell viability and increases ALOX15 expression in the HG‐injured model, consistent with the RNA‐seq results.

### 4.4 ALOX15 Promotes Neuronal Ferroptosis via Lipid Peroxidation

**FIGURE 4:**
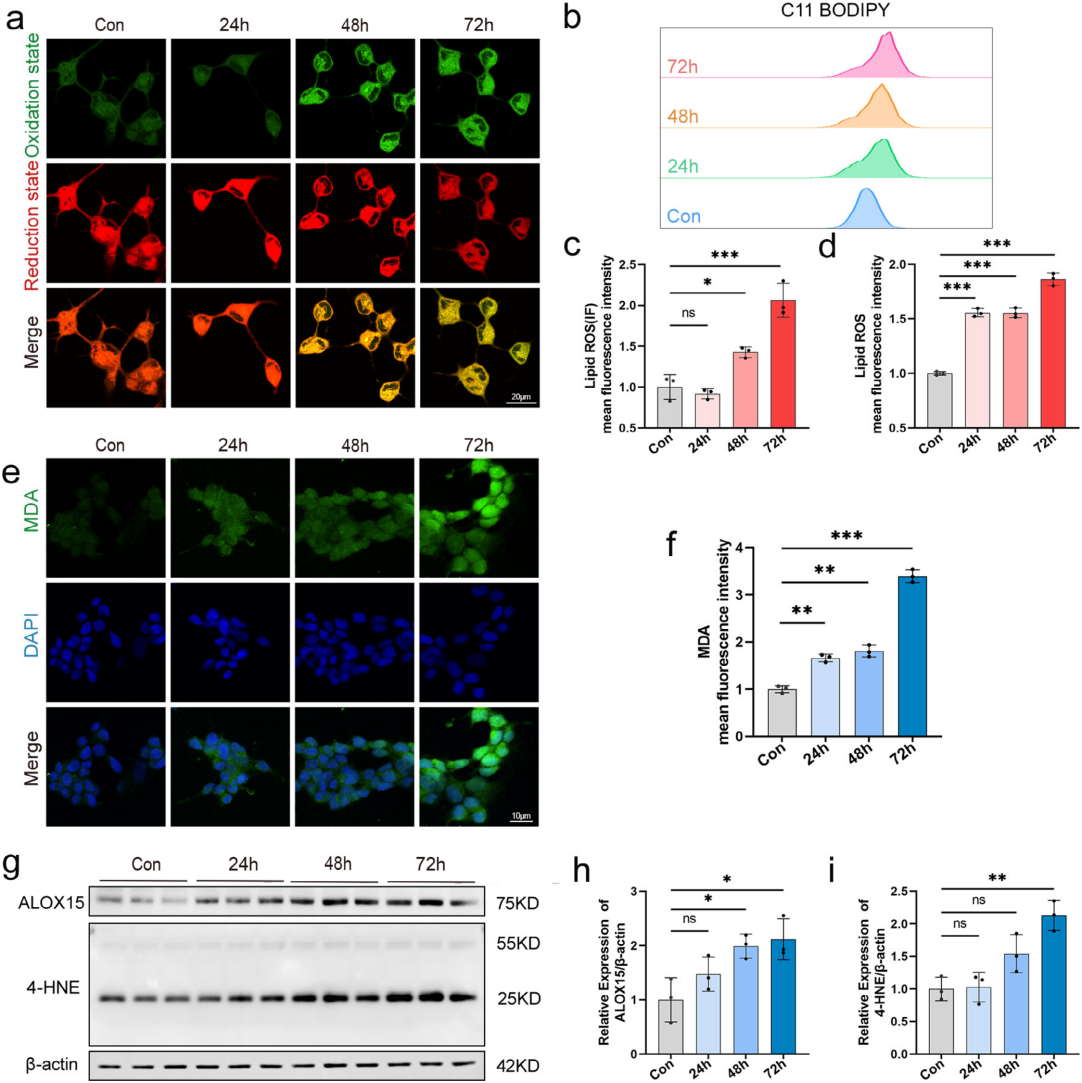
ALOX15 promotes neuronal ferroptosis via lipid peroxidation. Cells were treated with 50 mM glucose for 24, 48, and 72 h, respectively. (a) Representative images of live cell staining using C11‐BODIPY: Oxidized state (green), reduced state (red). Scale bar: 20 μm. (b) Flow cytometry detection of lipid ROS levels by C11‐BODIPY staining. (c) Quantitative data of lipid ROS detected by confocal fluorescence. (d) Quantitative data of lipid ROS detection by flow cytometry. (e) Representative images of MDA (green) immunofluorescence staining, DAPI staining (blue) for nucleic acid detection. Scale bar: 10 μm. (f) Quantitative data of MDA detected by confocal fluorescence. (g) Representative WB of ALOX15 and 4HNE. (h, i) Quantitative analysis of ALOX15 and 4HNE expression. Data are shown as mean ± SD. One‐way ANOVA was used, followed by Tukey's multiple comparisons using multiple sample means compared two‐by‐two. *p < 0.05, **p < 0.01, ***p < 0.001, ns *p* > 0.05. *n* = 3 independent cell culture preparations.

To explore the influence of HG on SH‐SY5Y cells lipid peroxidation levels, we used the C11 BODIPY probe for staining and flow cytometry assay (Figure 4a–d). As shown in Figure 3a, the oxidation state was stained by the probe with green fluorescence (488/510 nm), and the reduction state was stained with red fluorescence (581/591 nm). The results indicated that cellular lipid peroxidation increased under HG stimulation, while the reduction state decreased.

The change of MDA and 4‐HNE content represents the lipid peroxidation level [27]. Immunofluorescence results for MDA and WB results for 4‐HNE revealed that the expression of MDA and 4‐HNE in SH‐SY5Y cells from the HG group was higher than that in the control group, with a time‐dependent increase in expression (Figure 4e–i).

This evidence suggests that HG treatment not only increased the expression of ALOX15, but also increased the accumulation of lipid ROS, which promoted ferroptosis and impaired SH‐SY5Y cell viability.

### 4.5 Ferroptosis‐Related Proteins Are Activated in a HG Injury Cell Model

**FIGURE 5:**
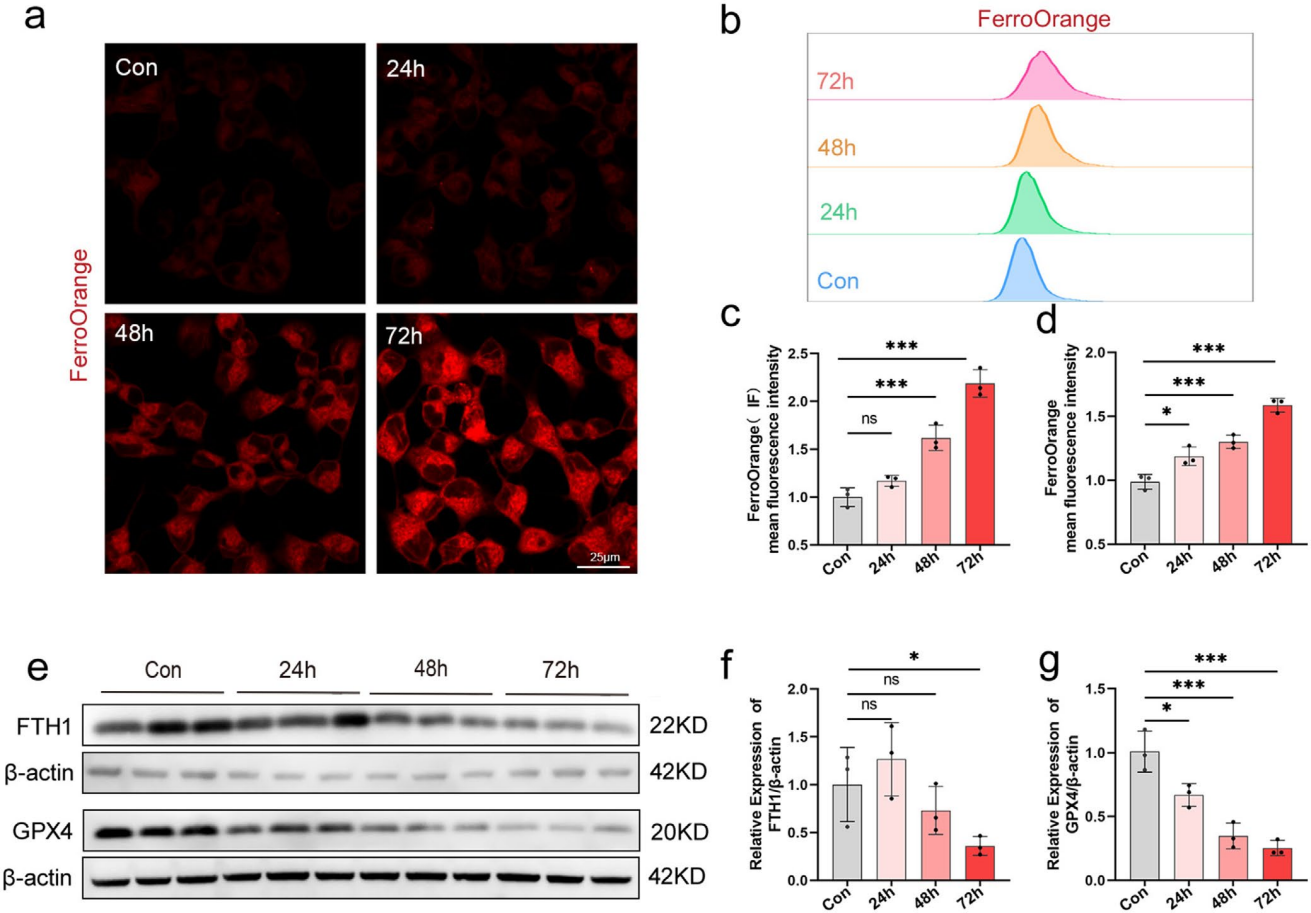
Ferroptosis‐related proteins are activated in a high glucose injury cell model. Fe^2+^ levels were detected by immunofluorescence and flow cytometry after treatment with 50 mM glucose for 24, 48, and 72 h, respectively. (a) Representative confocal fluorescence images of Fe^2+^ levels in the cells shown. Scale bar size is 25 μm. (b) Flow cytometry detection of Fe^2+^ levels. (c) Quantitative data of Fe^2+^ detected by confocal fluorescence. (d) Quantitative data of Fe^2+^ detected by flow cytometry. (e) WB analysis of GPX4 and FTH1, using β‐actin as a normalization control. (f, g) Quantitative assessment of the expression of GPX4 and FTH1. Data are shown as mean ± SD. One‐way ANOVA was used, followed by Tukey's multiple comparisons using multiple sample means compared two‐by‐two. *p < 0.05, ***p < 0.001, ns *p* > 0.05. *n* = 3 independent cell culture preparations.

Iron is a specific marker of ferroptosis, and excess intracellular ferrous ions lead to lipid peroxidation via the Fenton reaction, causing toxicity and damage to membrane structures and triggering ferroptosis. The results from ferrous ion fluorescent probe staining and flow cytometry assay on SH‐SY5Y cells showed that HG treatment increased the accumulation of ferrous ions in SH‐SY5Y cells compared to the control group, with levels rising over time (Figure 5a–d).

Additionally, we noted alterations in the levels of ferroptosis‐related markers FTH1 and GPX4. Western blot analysis showed: The control group exhibited high expression levels of FTH1 and GPX4 (Figure 5e–g). However, after HG incubation, the expression of GPX4 and FTH1 progressively decreased. These findings suggest that ferroptosis‐related proteins are activated, the anti‐lipid oxidation system is inhibited, and intracellular iron homeostasis is imbalanced, leading to an increase in Fe^2+^ in a high‐glucose‐injured cell model.

The above results indicated that under HG culture, SH‐SY5Y cell viability decreased, ferrous ion content increased, lipid peroxidation increased, ferroptosis signature protein increased, and ferroptosis occurred in the cells. Our cellular model of HG injury was successfully established. According to the results of WB, CCK‐8, and immunodeficiency assays, we decided to select 50 mM glucose treatment for 72 h as the modeling condition for the subsequent HG injury experiments.

### 4.6 Knockdown of ALOX15 Attenuates HG‐Induced Impairment of Neuronal Viability

**FIGURE 6:**
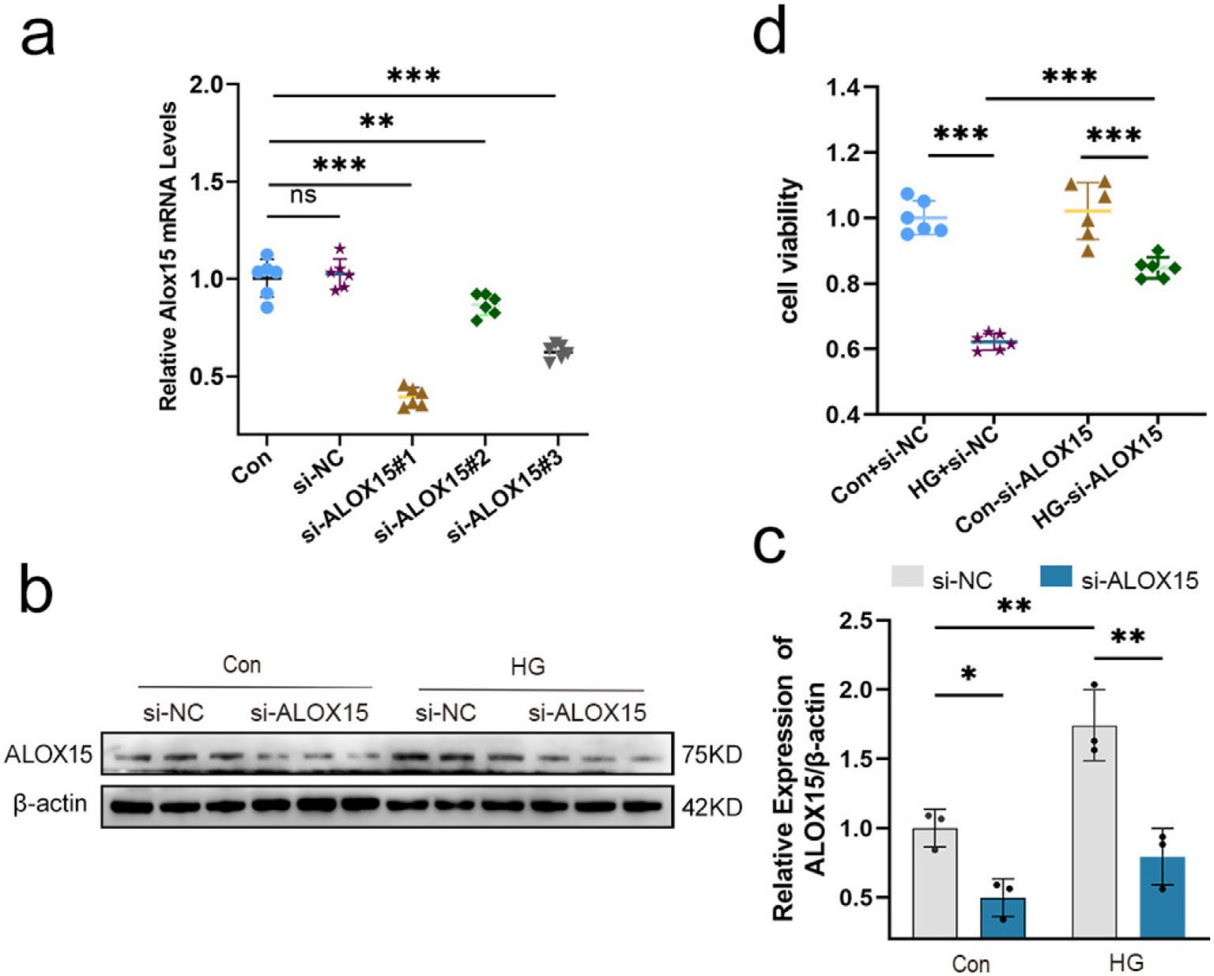
Knockdown of ALOX15 attenuates high glucose (HG)‐induced impairment of neuronal viability. Specific siRNA knockdown of ALOX15 was used and treated accordingly to grouping requirements. glucose: 50 mM. (a) ALOX15 expression was analyzed through qPCR, with mRNA levels normalized to β‐actin. (b) WB of ALOX15. (c) Measurement of ALOX15 expression levels. (d) CCK‐8 results of control and high glucose groups after ALOX15 knockdown. Data are shown as mean ± SD. One‐way ANOVA was used, followed by Tukey's multiple comparisons using multiple sample means compared two by two. *p < 0.05, **p < 0.01, ***p < 0.001. *n* = 3–6 independent cell culture preparations.

To assess the effect of ALOX15 on SH‐SY5Y cell damage caused by HG, we next performed siRNA transfection of SH‐SY5Y cells to knock down ALOX15. Initially, we used qPCR to evaluate ALOX15 knockdown efficiency, showing that the si‐ALOX15#1 sequence was knocked down most efficiently (Figure 6a). Consequently, we chose the si‐ALOX15#1 sequence for further transfections and performed WB experiments to verify the knockdown efficiency. The results demonstrated that ALOX15 expression in the knockdown group was notably reduced compared to the control group (Figure 6b,c). To explore whether the knockdown of ALOX15 could alleviate the influence of HG regarding SH‐SY5Y cell viability, we used CCK8 to detect cell viability in the control and HG groups. The results showed that SH‐SY5Y cell viability decreased in the HG group compared to the control group (Figure 6d). Of interest, however, the SH‐SY5Y cell viability of the HG + si‐ALOX15 group was significantly higher than that of the HG + si‐NC group.

These results suggest that our ALOX15 knockdown was effective and that the knockdown of ALOX15 attenuated HG‐induced impairment of SH‐SY5Y cell viability.

### 4.7 Knockdown of ALOX15 Inhibits SH‐SY5Y Cells Ferroptosis by Attenuating Lipid ROS Levels

**FIGURE 7:**
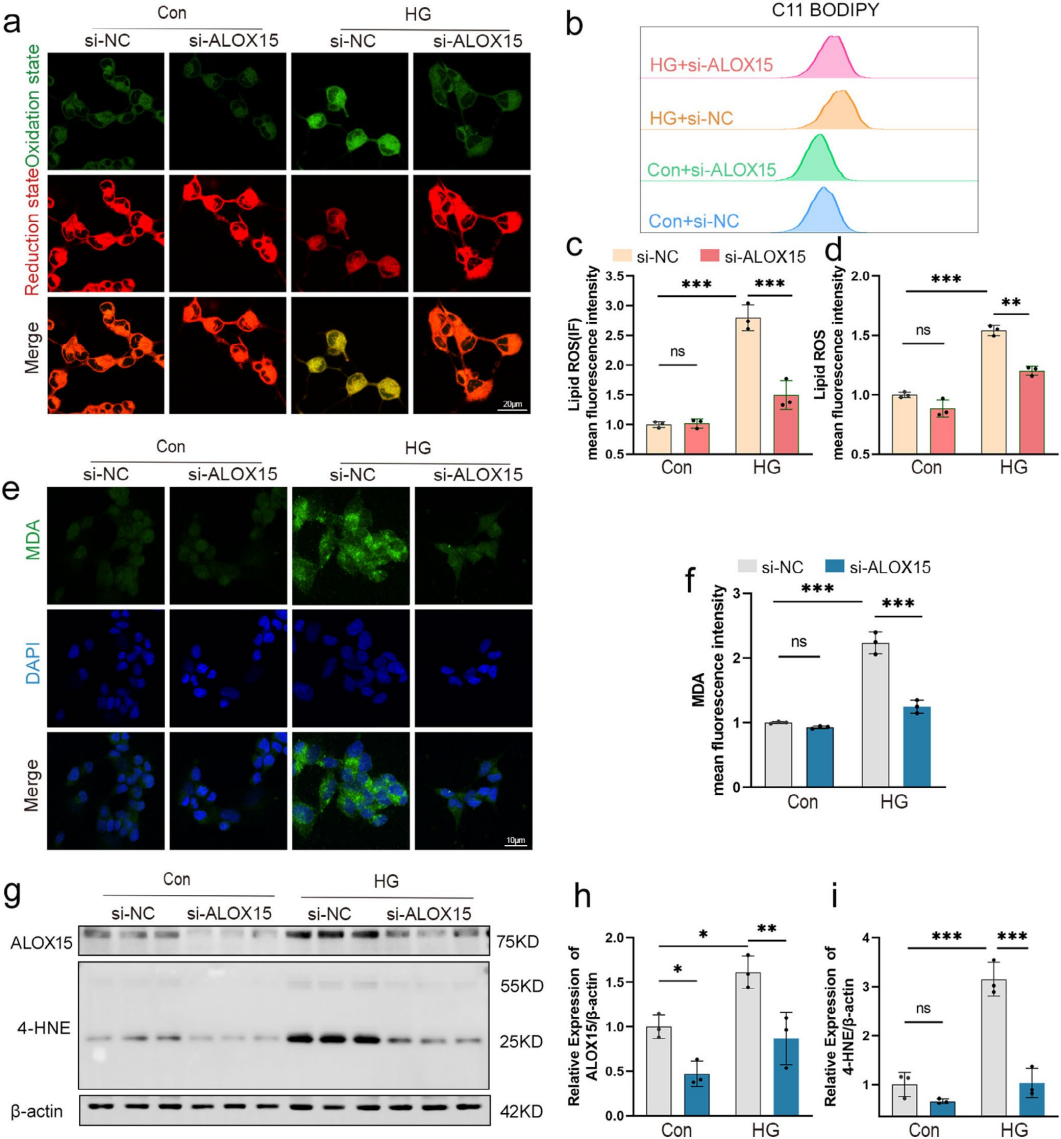
Knockdown of ALOX15 inhibits neuronal ferroptosis by attenuating lipid ROS levels. (a) Representative images of live cell staining using C11‐BODIPY: Oxidized state (green), reduced state (red). Scale bar: 20 μm. (b) Flow cytometry detection of lipid ROS levels by C11‐BODIPY staining. (c) Quantitative data of lipid ROS detected by confocal fluorescence. (d) Quantitative data of lipid ROS detection by flow cytometry. (e) Representative images of MDA (green) immunofluorescence staining, DAPI staining (blue) for nucleic acid detection. Scale bar: 10 μm. (f) Quantitative data of MDA detected by confocal fluorescence. (g) Representative WB of ALOX15 and 4HNE. (h, i) Quantitative analysis of ALOX15 and 4HNE expression. Data are shown as mean ± SD. One‐way ANOVA was used, followed by Tukey's multiple comparisons using multiple sample means compared two‐by‐two. *p < 0.05, **p < 0.01, ***p < 0.001, ns *p* > 0.05. *n* = 3 independent cell culture preparations.

To determine whether knockdown of ALOX15 protects against SH‐SY5Y cell injury by inhibiting lipid peroxidation, we observed the C11 BODIPY fluorescent probe staining method alongside a flow cytometry assay. We found that ALOX15 knockdown significantly inhibited the level of lipid peroxidation in HG‐induced SH‐SY5Y cells (Figure 7a–d).

Immunofluorescence results for MDA and WB for 4‐HNE showed that the levels of both MDA and 4‐HNE were increased in the HG group compared to the control group (Figure 7e–i). However, the expression of MDA and 4‐HNE in the HG + si‐ALOX15 group was significantly lower than in the HG + si‐NC group. These results indicate that knockdown of ALOX15 can effectively alleviate the degree of lipid peroxidation within cells under HG conditions, suggesting that ALOX15 plays an important role in lipid peroxidation regulation.

### 4.8 Knockdown of ALOX15 Attenuates HG‐Induced SH‐SY5Y Cells Ferroptosis

**FIGURE 8:**
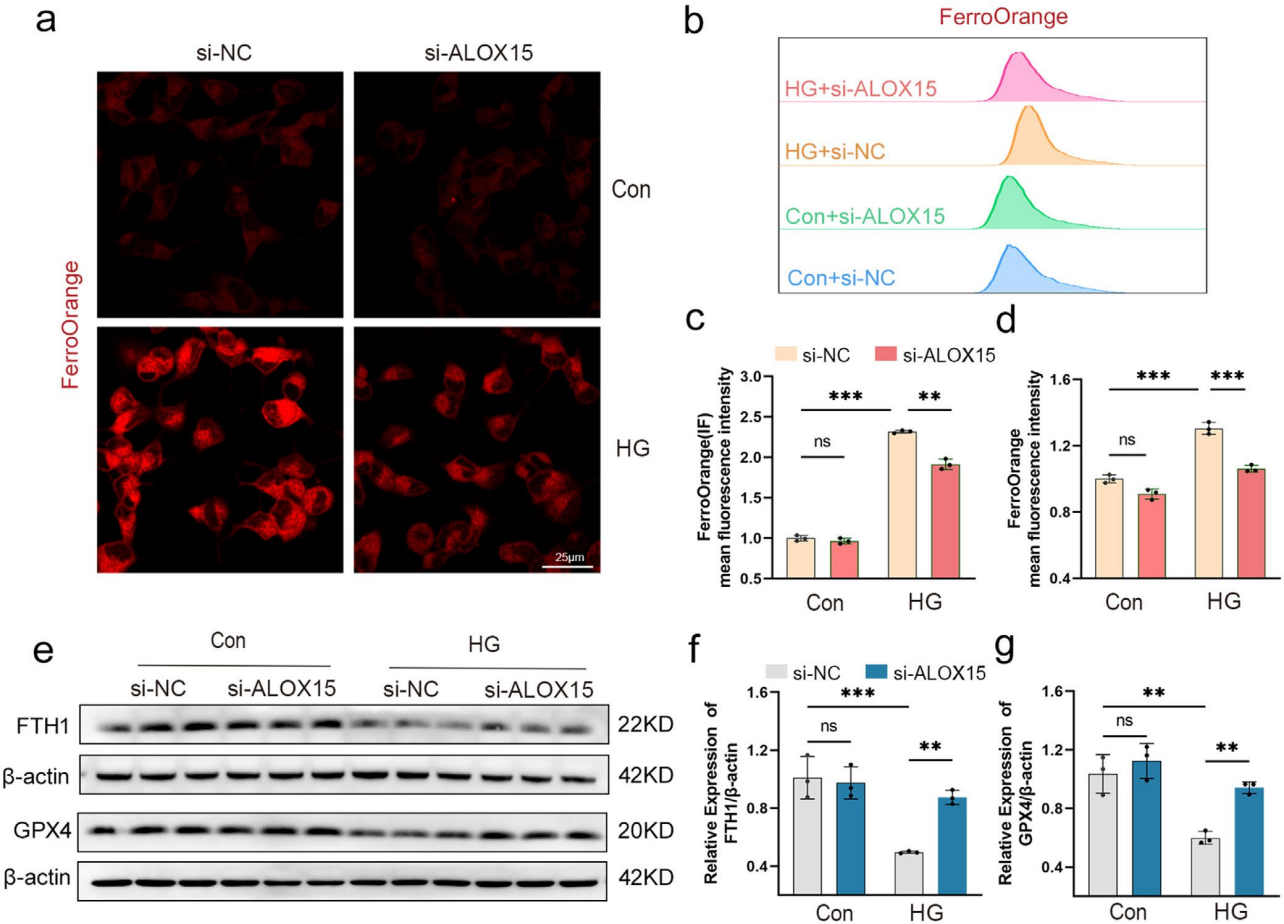
Knockdown of ALOX15 attenuates high glucose‐induced neuronal ferroptosis. (a) Representative confocal fluorescence images of Fe^2+^ levels in the cells shown. Scale bar size is 25 μm. (b) Flow cytometry detection of Fe^2+^ levels. (c) Quantitative data of Fe^2+^ detected by confocal fluorescence. (d) Quantitative data of Fe^2+^ detected by flow cytometry. (e) WB analysis of GPX4 and FTH1, using β‐actin as a normalization control (three samples in each group). (f, g) Quantitative assessment of the expression of GPX4 and FTH1. Data are shown as mean ± SD. One‐way ANOVA was used, followed by Tukey's multiple comparisons using multiple sample means compared two‐by‐two. **p < 0.01, ***p < 0.001, ns *p* > 0.05. *n* = 3 independent cell culture preparations.

The results of ferrous ion fluorescence probe staining and flow cytometry on SH‐SY5Y cells showed that the ferrous ion content of the HG group increased compared to the control group (Figure 8a–d). However, the elevation of ferrous ion fluorescence intensity in the HG + si‐ALOX15 group was smaller than in the HG + si‐NC group. This proved that SH‐SY5Y cells showed a large accumulation of ferrous ions after HG stimulation, and knockdown of ALOX15 could mitigate this phenomenon.

Additionally, we observed changes in the expression of the ferroptosis markers GPX4 and FTH1. WB analysis showed that the expression of GPX4 and FTH1 was decreased in the HG group compared to the control group (Figure 8e–g). However, the decrease in GPX4 and FTH1 was smaller in the HG + si‐ALOX15 group than in the HG + si‐NC group. This evidence suggests that ALOX15 knockdown mitigated ferroptosis in HG‐stimulated SH‐SY5Y cells.

## 5 Discussion

RF, as an ensemble learning algorithm, makes predictions by relying on decision trees and is helpful for identifying key genes [28, 29]. RF methods have been beneficial for various diseases such as diabetes and breast cancer [30, 31, 32]. We innovatively employed the RF algorithm to model and analyze gene expression data and accurately identified ALOX15 as a key candidate gene for DPNP ferroptosis through feature importance scoring. Additionally, as there are currently no literature reports on ferroptosis and DPNP, we established both DPNP mouse models and high‐glucose‐induced SH‐SY5Y cell injury models to provide experimental evidence for the connection between DPNP and ferroptosis. Strikingly, we found that ALOX15 was significantly upregulated in DRG tissue of DPNP mouse and was associated with ferroptosis biomarkers.

Ferroptosis, as a novel cell death mechanism, involves three main factors: an increase in intracellular free iron, depletion of glutathione/GPX4, and peroxidation of membrane PUFA [33, 34, 35]. Growing evidence suggests that pathophysiologic changes in ferroptosis are observed in a variety of neuropathic pain and diabetes and its complications [11]. Controlling ferroptosis has become a potential treatment for neuropathic pain [12, 36]. For instance, Kexing Wan et al. demonstrated that electroacupuncture alleviates neuropathic pain by suppressing ferroptosis in DRG through inhibition of the SAT1‐ALOX15 signaling axis [16]. Our research shows that ALOX15 is a key regulator of ferroptosis and is related to high‐glucose‐induced SH‐SY5Y cell toxicity, manifested as intracellular free iron accumulation, downregulation of FTH1, GPX4 expression, and elevated lipid peroxidation markers such as MDA and 4‐HNE. It is notable that ALOX15 knockdown significantly attenuated high‐glucose‐induced ferroptosis, preserved GPX4‐mediated antioxidant capacity, and restored cellular viability, highlighting its therapeutic potential for DPNP.

ALOX15, as a member of the lipoxygenase family, is mainly responsible for catalyzing the oxidation of various fatty acids to produce multiple lipid components, which contribute to the pathophysiological processes of many diseases [37, 38, 39, 40, 41]. Since neural tissues contain high levels of PUFA, lipid peroxidation is likely a primary characteristic of ferroptosis in DPNP patients [42, 43, 44]. As a driver of ferroptosis, high levels of lipid peroxidation disrupt cellular functions [34]. Numerous products of lipid peroxidation are chemically reactive aldehydes, including MDA, 4‐hydroxy‐2‐nonenal (4‐HNE), and acrolein, among others [45, 46], with various cytotoxic effects. We found that ferroptosis shows “biphasic regulation” characteristics in DPNP: on the one hand, the HG environment increases the free iron pool through oxidative stress; on the other hand, ALOX15 upregulation accelerates PUFA oxidation, generating cytotoxic aldehydes (e.g., 4‐HNE, MDA) that disrupt membrane integrity and culminate in cell death. Further studies have found that after specific knockdown of ALOX15 by siRNA, the survival rate of SH‐SY5Y cells induced by HG increased to 85%, and the level of ferroptosis was inhibited. This is consistent with the mechanism reported by Cai et al. that baicalein prevents ferroptosis by chelating iron and interacting with ALOX15, thereby alleviating lung injury [47]. This mechanism may explain why traditional analgesic drugs have limited efficacy in the treatment of neuropathic pain, while targeted intervention against ALOX15 shows more significant protective effects.

However, our study has several limitations. First, we only used one data set to construct the model, which may not provide a sufficient sample size for the RF approach. Future studies should include more datasets to verify the model's reliability. Second, we have yet to knock out ALOX15 in the DPNP animal model to further validate our findings. Thus, these limitations need to be addressed in future studies.

## 6 Conclusions

Overall, our results confirm that ALOX15 expression is significantly associated with DPNP. More importantly, we found that ALOX15 contributes to the development of DPNP by promoting neuronal ferroptosis, whereas knockdown of ALOX15 inhibited the onset of neuronal ferroptosis and restored neuronal viability in DPNP. In addition, these findings highlight ALOX15 as an important factor in ferroptosis for DPNP treatment, support its identification as a DPNP biomarker, and offer new targets and strategies for treating ferroptosis‐related diseases, especially DPNP.

## Author Contributions

Zhongjie Liu conceived and designed the research and drafted and revised the manuscript. Zhiye Feng performed the experiments. Fuye Li helped to analyze the data and interpreted the results of the experiments. Zhiqiang Lin was responsible for the animal experiments. Jian Liu, Xi Chen, and Wenxu Yan provided experimental help. All authors approved the final edited version of the manuscript.

## Conflicts of Interest

The authors declare no conflicts of interest.

## Supporting information


Data S1.

## Data Availability

All the data supporting the findings of this study are available within the article and from the corresponding author upon reasonable request.
