# Supplementary material for: ALOX15‐Mediated Neuron Ferroptosis Was Involved in Diabetic Peripheral Neuropathic Pain
Source: CNS Neurosci Ther. 2025 May 19;31(5):e70440. doi: 10.1111/cns.70440 (PMC12087304; doi:10.1111/cns.70440)

Full unedited gel/blot for Figure 2

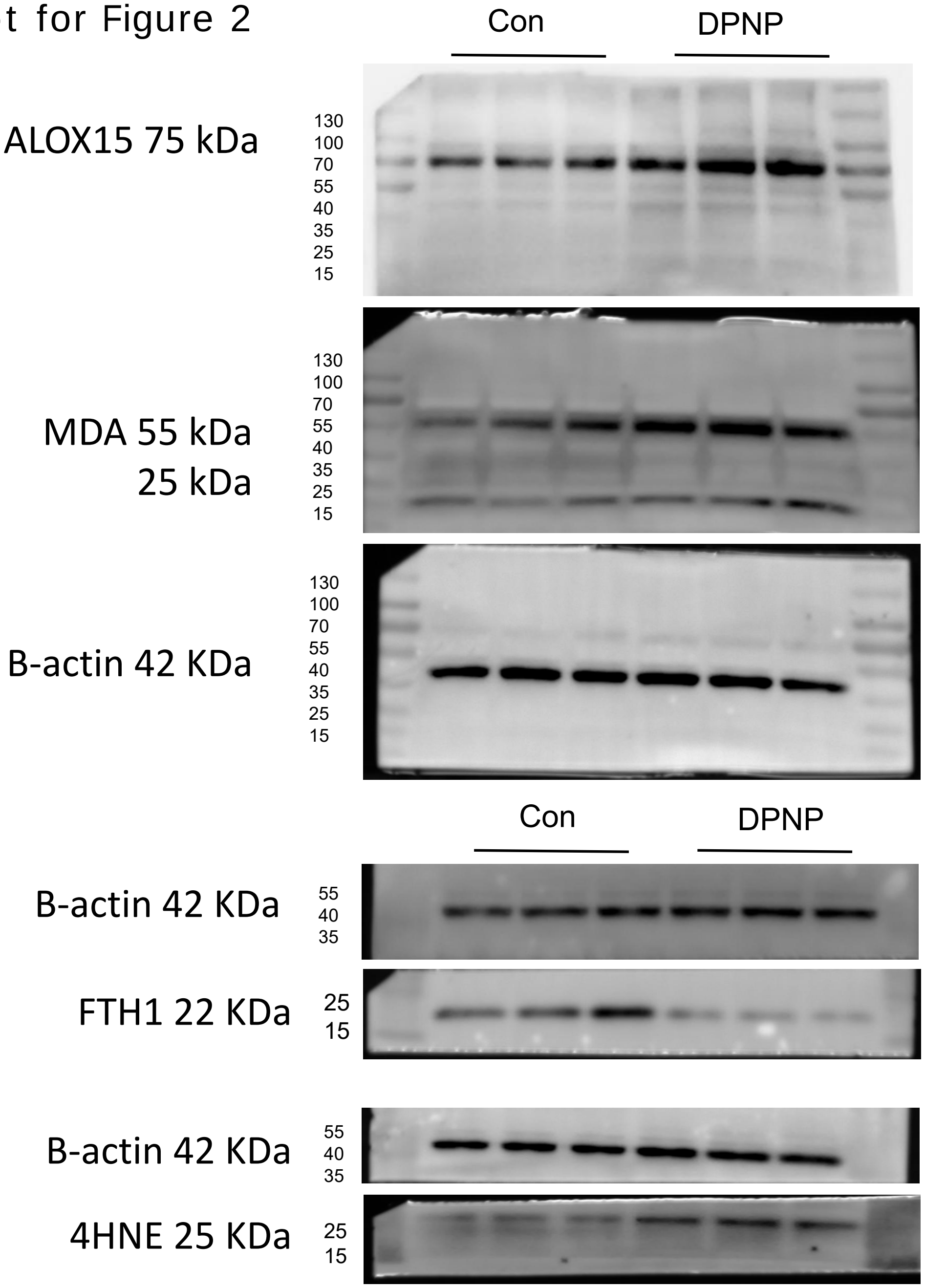

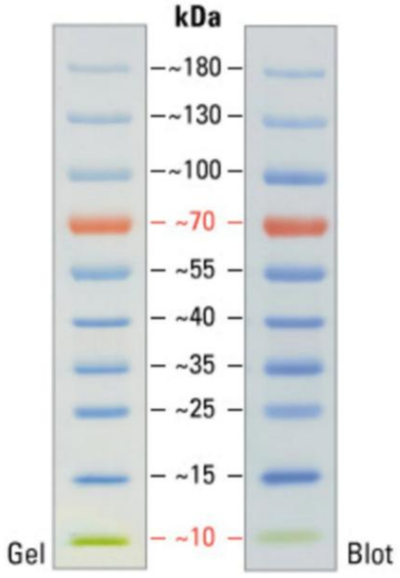

#26616

Full unedited gel/blot for Figure 3

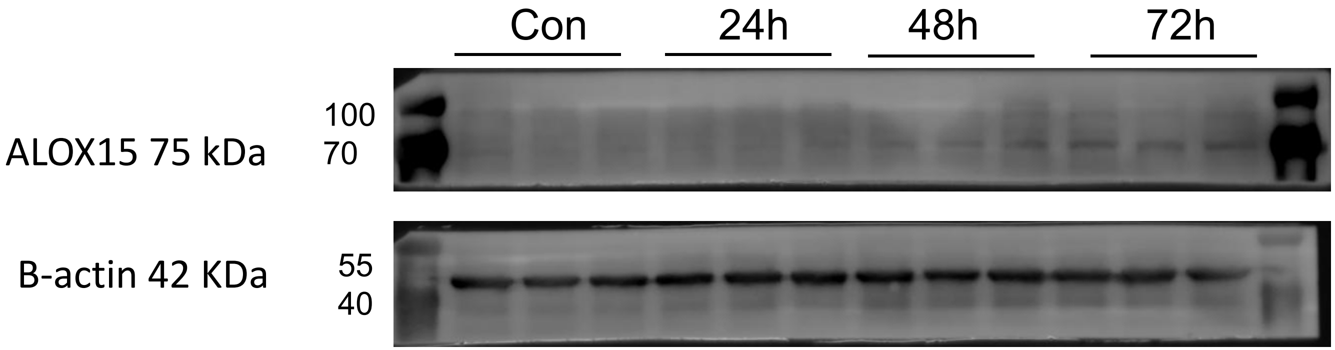

Full unedited gel/blot for Figure 4

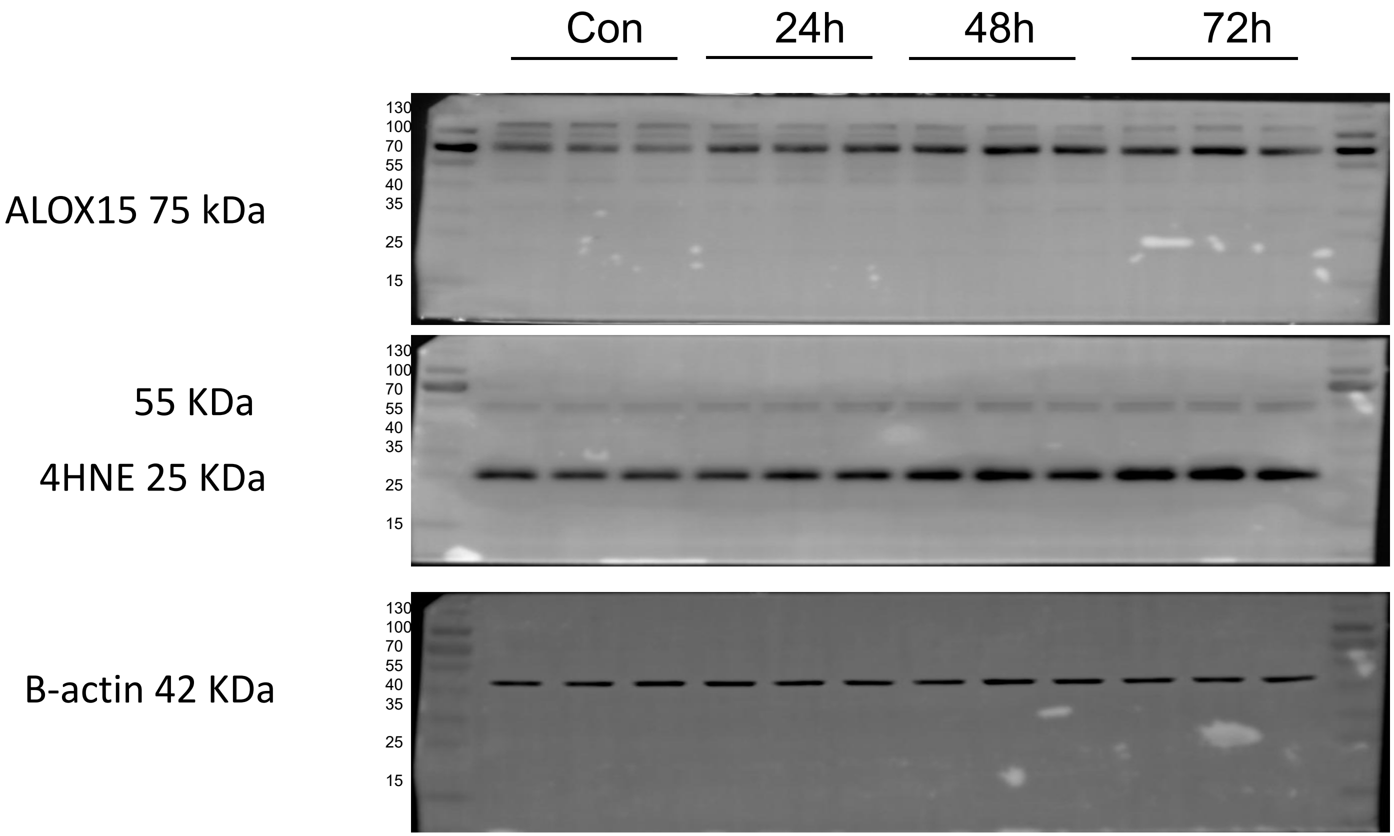

Full unedited gel/blot for Figure 5

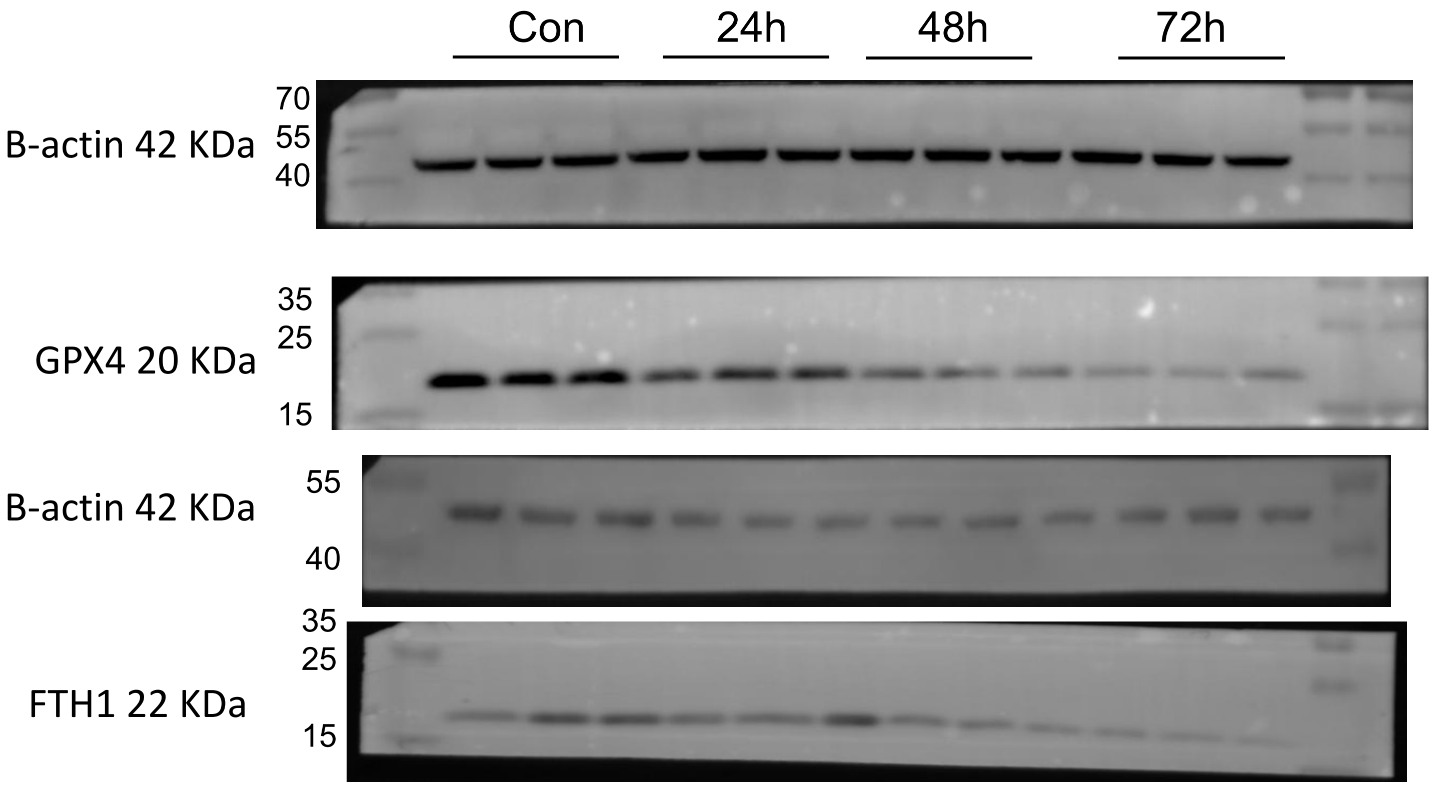

Full unedited gel/blot for Figure 6

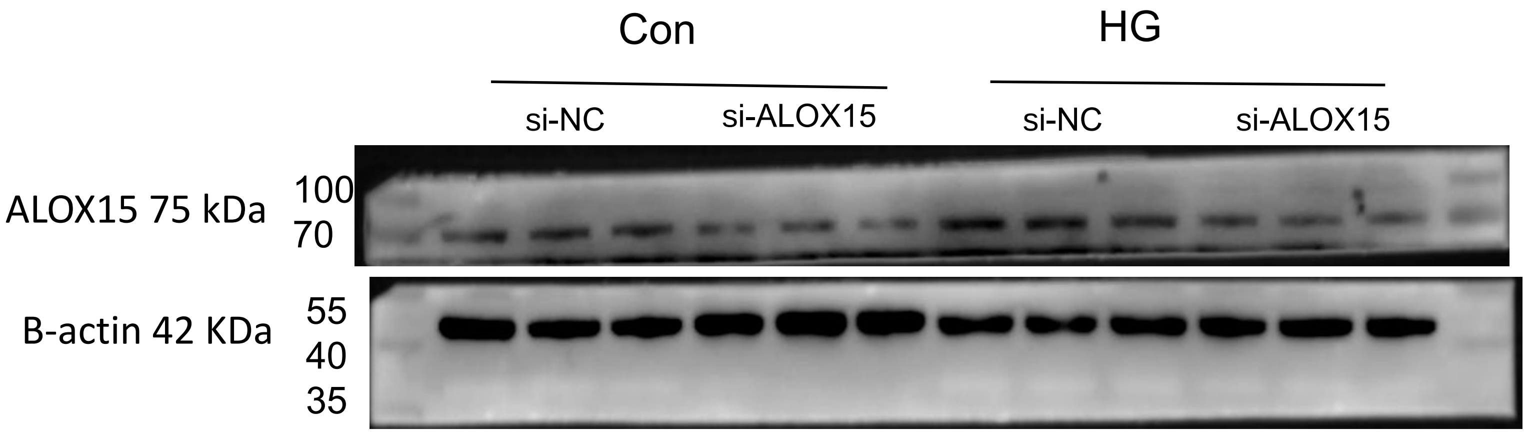

Full unedited gel/blot for Figure 7

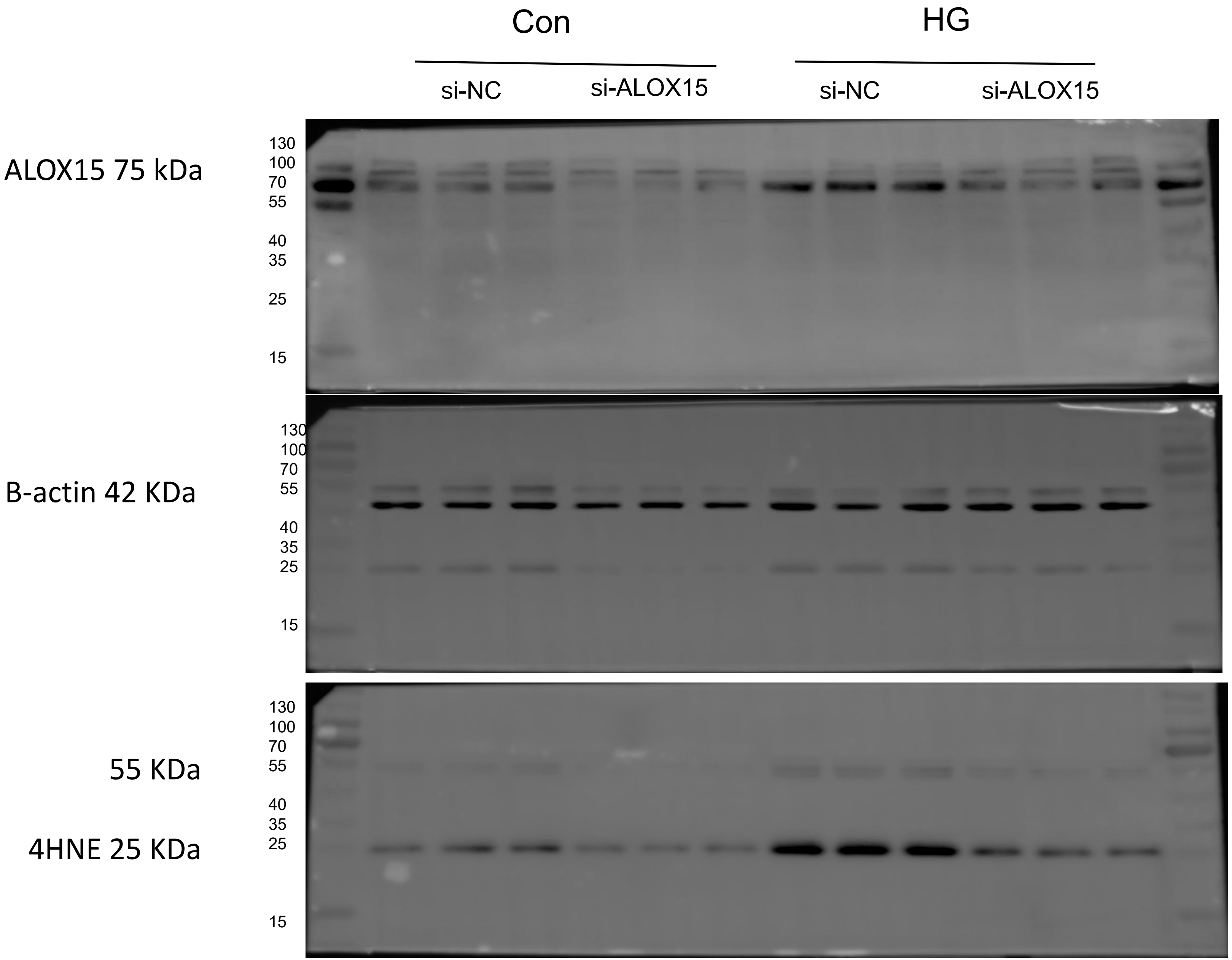

Full unedited gel/blot for Figure 8

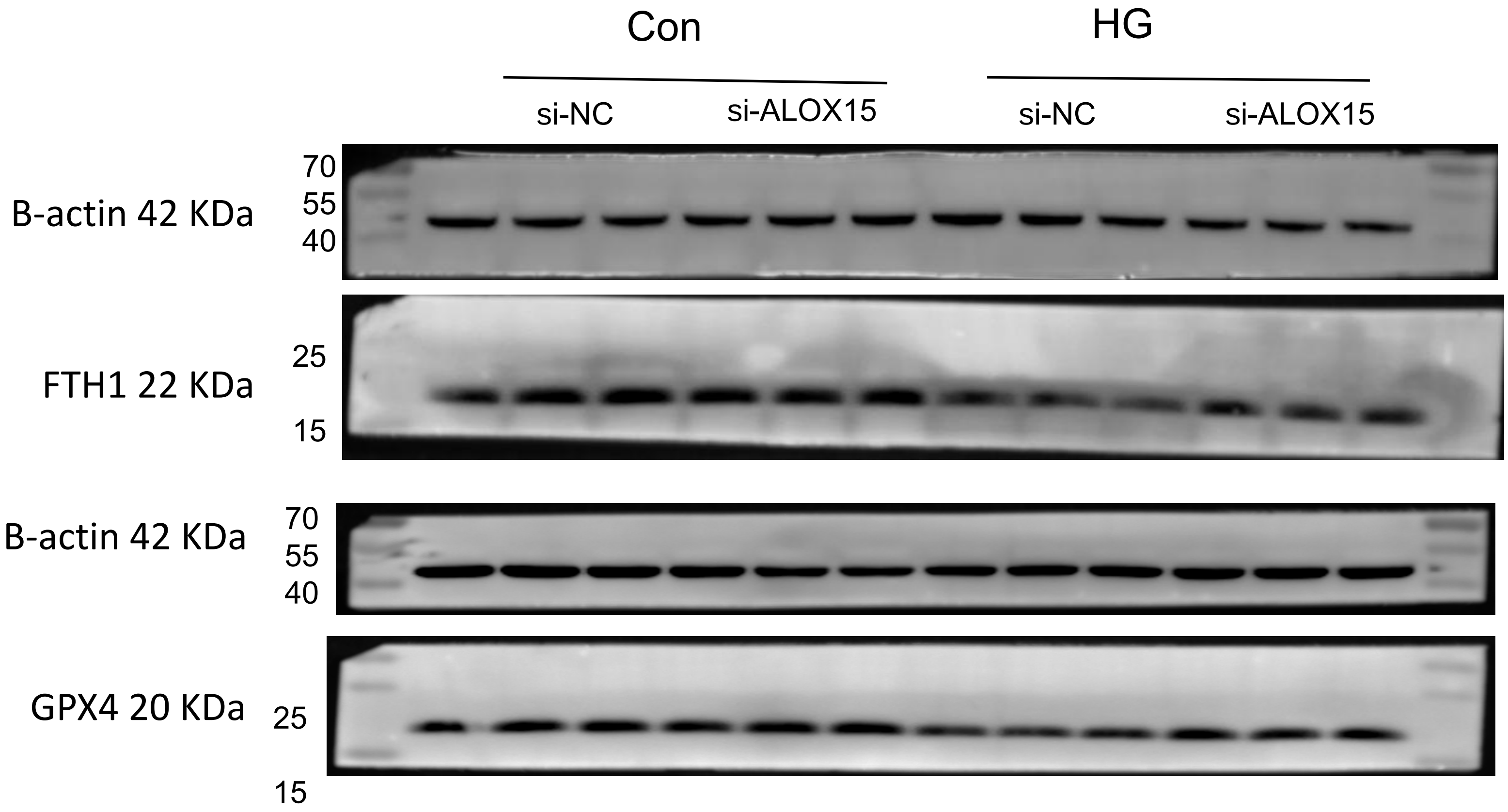

Supplement: Supplementary file 1 — Data S1. [file CNS-31-e70440-s001.zip › Supplemental Files.pdf]
